# Supplementary material for: Predictive Utility and Metabolomic Signatures of TG/HDL-C Ratio for Metabolic Syndrome Without Cardiovascular Disease and/or Diabetes in Qatari Adults
Source: Metabolites. 2025 Aug 28;15(9):574. doi: 10.3390/metabo15090574 (PMC12471751; doi:10.3390/metabo15090574)
Supplement: Supplementary file 1 [file metabolites-15-00574-s001.zip › metabolites-3769034_Supplementary_Table_Cycle_2_Revision.pdf]

Diagnostic Performance and Metabolomic Signatures of 2 TG/HDL-C Ratio for Metabolic Syndrome Without Cardiovascular Disease and/or Diabetes in Qatari Adults

Noora Kano[1*], Najeha Anwardeen [2], Khaled Naja [2], Asma ElAshi [2], and Mohamed A. Elrayess [2,3*]

[1] Military Medical City Hospital, Medical Services, Qatar Armed Forces, Qatar

[2] Biomedical Research Center, QU Health, Qatar University, Doha P.O. Box 2713, Qatar

[3]College of Medicine, QU Health, Qatar University, Doha P.O Box 2713, Qatar

* Author to whom correspondence should be addressed: m.elrayess@qu.edu.qa ; no.kano@mmch.qa (N.K.)

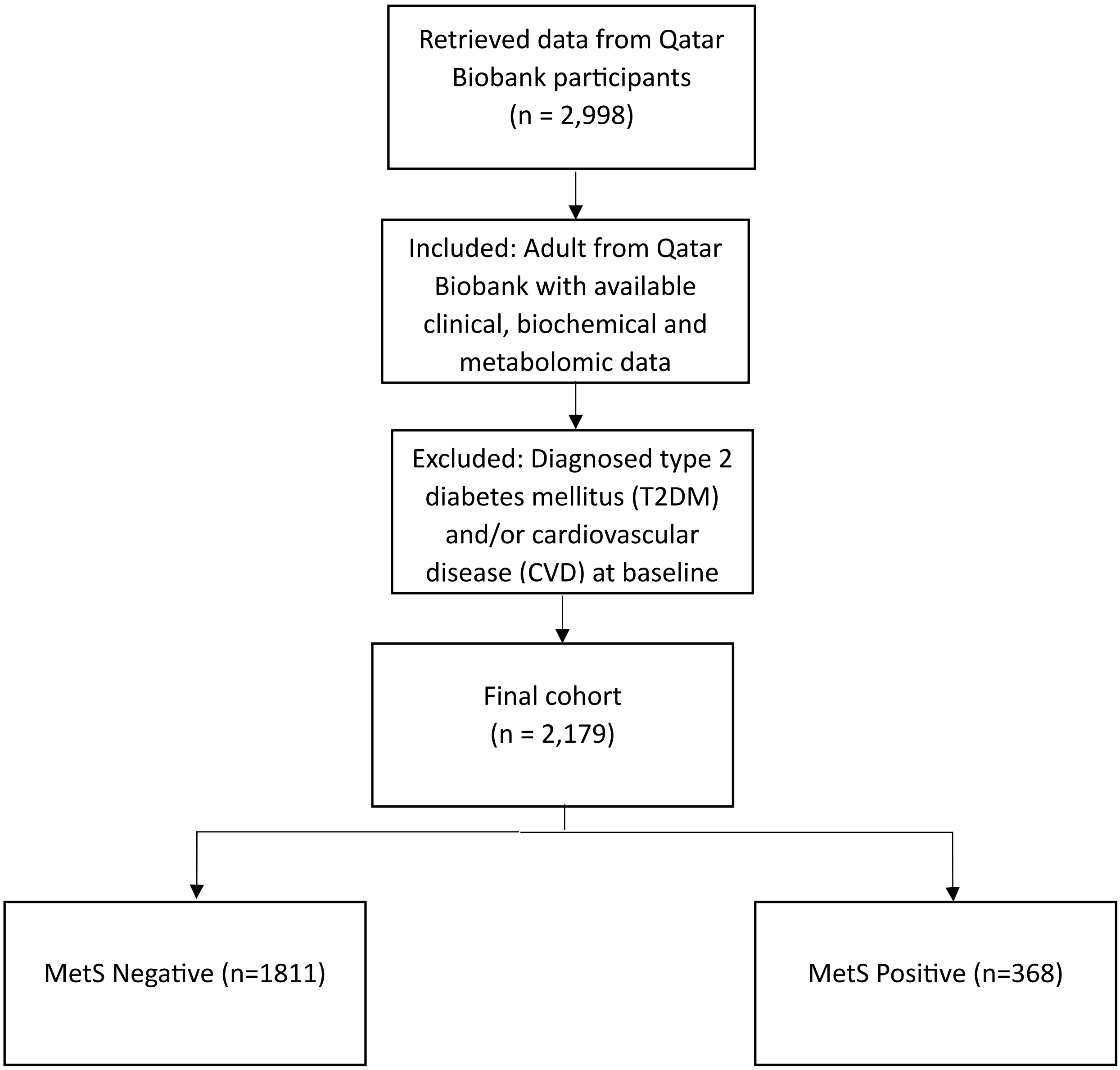

Supplementary Figure S1. Flowchart illustrating the selection of study participants from Qatar Biobank. Data from 2,998 participants with available metabolomics data were retrieved. Individuals with a diagnosis of type 2 diabetes mellitus (T2DM) and/or cardiovascular disease (CVD) at baseline (n = 819) were excluded, resulting in a final analytic cohort of 2,179 participants.

Supplementary Table S1: Categorical characteristics of participants stratified by metabolic syndrome status.

	**MetS negative**	**MetS positive**	**p-value**
Gender (male/female)	870/941	225/143	<0.0001
BMI (≥ 30 kg/m²; yes/no)	1112/699	315/53	<0.0001
Central obesity (no/yes)	1270/538	103/263	<0.0001
Elevated Triglyceride (no/yes)	1605/206	108/260	<0.0001
Elevated fasting blood glucose (no/yes)	1656/155	182/186	<0.0001
Elevated blood pressure (no/yes)	1759/52	267/101	<0.0001
Reduced HDL (no/yes)	1136/675	36/332	<0.0001

Supplementary Table S2: General characteristics of participants

		MetS Negative (n=1811)	MetS Positive (n=368)	p-value
**General Characteristics**	Age	33 (27-43)	42 (34-50)	<0.001
	Systolic blood pressure (mmHg)	109 (101-118)	120.5 (111-133)	<0.001
	Diastolic blood pressure (mmHg)	71 (65-77)	79 (71-87)	<0.001
**Lipid profile**	TG/HDL-C	0.72 (0.47-1.08)	1.8 (1.32-2.62)	<0.001
	TC/HDL-C	3.46 (2.84-4.14)	4.83 (4.23-5.74)	<0.001
	LDL-C/HDL-C	2.1 (1.58-2.7)	3.04 (2.44-3.7)	<0.001
	NonHDL-C/HDL-C	2.46 (1.84-3.14)	3.83 (3.23-4.74)	<0.001
	NonHDL-C (mmol/L)	3.4 (2.84-4)	4.14 (3.55-4.73)	<0.001
	TG (mmol/L)	1 (0.73-1.34)	2 (1.4-2.58)	<0.001
	HDL-C(mmol/L)	1.39 (1.18-1.63)	1.06 (0.94-1.19)	<0.001
	LDL-C Calc (mmol/L)	3 (2.38-3.46)	3.17 (2.66-3.89)	<0.001
	TC (mmol/L)	4.8 (4.3-5.4)	5.2 (4.6-5.8)	<0.001
**Blood Sugar**	FBG (mmol/L)	4.9 (4.6-5.2)	5.6 (5-5.9)	<0.001
	Insulin (uU/mL)	8.1 (6-13)	17 (11.95-30)	<0.001
	C-Peptide (ng/mL)	2 (1.49-2.77)	3.33 (2.56-4.93)	<0.001
	HbA1C (%)	5.3 (5.1-5.5)	5.6 (5.3-5.8)	<0.001
**Insulin resistance**	TyG index	8.26 (7.95-8.58)	9.04 (8.79-9.32)	<0.001
**Anthropometric Measures**	BMI (Kg/m^2)	27 (23.85-30.48)	31.83 (28.66-35.23)	<0.001
	Weight (Kg)	74.3 (63.9-85.05)	88.8 (75.9-100.43)	<0.001
	waist size (cm)	84 (76-93)	98 (88-106)	<0.001
	hips size (cm)	104 (98-111)	111 (104.25-118)	<0.001
	waist to hip ratio	0.81 (0.74-0.88)	0.88 (0.82-0.94)	<0.001
**Liver function**	Alkaline Phosphatase (U/L)	66 (55-78)	70 (59-83)	<0.001
	ALT (U/L)	17 (12-26)	24 (18-33)	<0.001
	AST (U/L)	18 (15-21)	19 (16-23)	<0.001
	Bilirubin Total (μmol/L)	6.4 (5-9)	5.65 (4.2-8)	<0.001
	Albumin (g/L)	46 (44-47)	45 (43-47)	<0.001
**Kidney Function**	Creatinine (μmol/L)	65 (55.5-78)	70.5 (58-80)	<0.001
	Uric Acid (μmol/L)	285 (234-341)	333 (273-393.25)	<0.001
**Cardiac Function**	NT-proBNP (pg/mL)	24.4 (13-40.8)	19.4 (11.6-39.92)	<0.05
	Homocysteine (μmol/L)	8.25 (6.8-10.07)	8.3 (7-10)	0.77
**Thyroid Function**	Free Thyroxine (pmol/L)	13.2 (12.25-14.29)	12.86 (12-13.8)	<0.001
	Free Triiodothyronine (pmol/L)	4.47 (4.07-4.8)	4.6 (4.2-4.9)	<0.01
	Thyroid Stimulating Hormone (mIU/L)	1.4 (0.98-2.07)	1.42 (0.95-2.14)	0.8239

All variables are presented as median (interquartile range). Comparisons between groups were performed using the Mann–Whitney U test. A p-value < 0.05 was considered statistically significant. Abbreviations: BMI, body mass index; HbA1C, glycated hemoglobin; HDL, high-density lipoprotein; LDL, low-density lipoprotein; NT-proBNP, N-terminal pro ‑ B-type natriuretic peptide; ALT, alanine transaminase; AST, aspartate aminotransferase; FBG, Fasting Blood Glucose

Supplementary Table S3: Gender-stratified logistic regression results for the association between lipid ratios and Metabolic Syndrome.

Males										
	Unadjusted					Adjusted				
	OR	95% CI	AUC	AUC 95% CI	p	OR	95% CI	AUC	AUC 95% CI	p
TG/HDL	2.74	2.32 – 3.26	0.838	0.811 - 0.867	<0.0001	3.00	2.48 – 3.69	0.904	0.885 - 0.923	<0.0001
TC/HDL	1.78	1.59 – 2.00	0.791	0.762 - 0.821	<0.0001	1.68	1.50 – 1.92	0.869	0.847 - 0.893	<0.0001
LDL/HDL	1.63	1.44 – 1.87	0.722	0.688 - 0.756	<0.0001	1.47	1.29 – 1.70	0.844	0.818 - 0.869	<0.0001
non-HDL/HDL	1.78	1.54 – 2.00	0.791	0.762 - 0.821	<0.0001	1.68	1.50 – 1.92	0.869	0.847 - 0.893	<0.0001

Females										
	Unadjusted					Adjusted				
	OR	95% CI	AUC	AUC 95% CI	p	OR	95% CI	AUC	AUC 95% CI	p
TG/HDL	36.3	21.3 – 62.9	0.907	0.880 - 0.935	<0.0001	3.43	3.15 – 8.78	0.922	0.897 - 0.947	<0.0001
TC/HDL	5.06	3.95 – 6.61	0.871	0.841 - 0.902	<0.0001	4.54	3.53 – 5.94	0.881	0.853 - 0.909	<0.0001
LDL/HDL	4.16	3.23 – 5.43	0.805	0.768 - 0.842	<0.0001	3.59	2.78 – 4.71	0.826	0.792 - 0.861	<0.0001
non-HDL/HDL	5.06	3.95 – 6.61	0.871	0.841 - 0.902	<0.0001	4.54	3.53 – 5.94	0.881	0.853 - 0.909	<0.0001

Abbreviations: AUC, area under the receiver operating characteristic curve; OR, odds ratio; CI, confidence interval; TG, triglyceride; HDL, high-density lipoprotein cholesterol; TC, total cholesterol; LDL, low-density lipoprotein cholesterol; Non-HDL, non–high-density lipoprotein cholesterol; BMI, body mass index. Adjusted models include age, sex, and BMI as covariates.

Supplementary Table S4: Sensitivity analysis of logistic regression models excluding BMI as a covariate.

	OR	95% CI	AUC	95% CI	p
TG/HDL	4.3	3.61 - 5.16	0.876	0.858 - 0.895	<0.0001
TC/HDL	2.19	1.97 - 2.44	0.838	0.818 - 0.859	<0.0001
LDL/HDL	1.96	1.74 - 2.22	0.785	0.761 - 0.809	<0.0001
non-HDL/HDL	2.19	1.97 - 2.44	0.838	0.818 - 0.859	<0.0001

Abbreviations: AUC, area under the receiver operating characteristic curve; OR, odds ratio; CI, confidence interval; TG, triglyceride; HDL, high-density lipoprotein cholesterol; TC, total cholesterol; LDL, low-density lipoprotein cholesterol; Non-HDL, non–high-density lipoprotein cholesterol; BMI, body mass index. Adjusted models include age and sex as covariates.

Supplementary Table S5: Logistic regression analysis of individual lipid markers and their association with MetS in stratified by gender.

Males										
	Unadjusted					Adjusted				
	OR	95% CI	AUC	95% CI	p	OR	95% CI	AUC	95% CI	p
TG	3.79	3.09 - 4.72	0.818	0.7865 - 0.8490	<0.0001	4.27	3.35 - 5.54	0.902	0.8824 - 0.9211	<0.0001
HDL-C	0.02	0.01 - 0.04	0.762	0.7307 - 0.7934	<0.0001	0.018	0.0076 - 0.0390	0.874	0.8522 - 0.8967	<0.0001
LDL-C	1.32	1.12 - 1.56	0.571	0.5299 - 0.6121	0.0012	1.1	0.91 - 1.34	0.825	0.7980 - 0.8526	<0.0001
non-HDL-C	1.89	1.62 - 2.20	0.692	0.6553 - 0.7278	<0.0001	1.72	1.44 - 2.05	0.841	0.8151 - 0.8675	<0.0001

Females										
	Unadjusted					Adjusted				
	OR	95% CI	AUC	AUC 95% CI	p	OR	95% CI	AUC	AUC 95% CI	p
TG	13.19	9.08 - 19.72	0.863	0.827 - 0.899	<0.0001	12.05	8.19 - 18.27	0.888	0.857 - 0.918	<0.0001
HDL-C	0.00166	0.00054 - 0.00468	0.875	0.844 - 0.905	<0.0001	0.00138	0.00041 - 0.00417	0.896	0.870 - 0.923	<0.0001
LDL-C	1.56	1.26 - 1.94	0.604	0.552 - 0.656	0.0005	1.33	1.06 - 1.67	0.721	0.678 - 0.763	<0.0001
non-HDL-C	2.33	1.91 - 2.87	0.722	0.677 - 0.768	<0.0001	2.06	1.67 - 2.56	0.768	0.728 - 0.808	<0.0001

Abbreviations: AUC, area under the receiver operating characteristic curve; OR, odds ratio; CI, confidence interval; TG, triglyceride; HDL, high-density lipoprotein cholesterol; TC, total cholesterol; LDL, low-density lipoprotein cholesterol; Non-HDL, non–high-density lipoprotein cholesterol; BMI, body mass index. Adjusted models include age, sex, and BMI as covariates.
